# Are severe musculoskeletal injuries associated with symptoms of common mental disorders among male European professional footballers?

**DOI:** 10.1007/s00167-015-3729-y

**Published:** 2015-08-02

**Authors:** Vincent Gouttebarge, Haruhito Aoki, Jan Ekstrand, Evert A. L. M. Verhagen, Gino M. M. J. Kerkhoffs

**Affiliations:** 1 Academic Center for Evidence based Sports medicine (ACES), Academic Medical Center, Amsterdam, The Netherlands; 2 Amsterdam Collaboration for Health and Safety in Sports (ACHSS), Amsterdam, The Netherlands; 3 Department of Orthopaedic Surgery, Academic Medical Center, Amsterdam, The Netherlands; 4 World Players’ Union (FIFPro), Scorpius 161, 2132 LR Hoofddorp, The Netherlands; 5 St. Marianna University School of Medicine, Kawasaki, Japan; 6 Department of Medical and Health Sciences, Linköping University, Linköping, Sweden; 7 Department of Public and Occupational Health, VU University Medical Center, Amsterdam, The Netherlands

**Keywords:** Soccer, Injuries, Surgeries, Mental disorders

## Abstract

**Purpose:**

To explore the associations of severe musculoskeletal injuries (joint and muscles) and surgeries with symptoms of common mental disorders (distress, anxiety/depression, sleeping disturbance, adverse alcohol behaviour , smoking, adverse nutrition behaviour) among male European professional footballers.

**Methods:**

Cross-sectional analyses were conducted on electronic questionnaires completed by professional footballers recruited from the national players’ unions of Finland, France, Norway, Spain or Sweden. The number of severe (time loss of more than 28 days) musculoskeletal injuries (total, joint, muscle) and surgeries during a professional football career was examined through four questions, while symptoms of common mental disorders were evaluated through validated scales.

**Results:**

A total of 540 professional footballers (mean age of 27 years; 54 % playing in the highest leagues) participated in the study. Sixty-eight per cent of the participants had already incurred one or more severe joint injuries and 60 % one or more severe muscle injuries. Prevalence of symptoms of common mental disorders ranged from 3 % for smoking to 37 % for anxiety/depression and 58 % for adverse nutrition behaviour. The number of severe musculoskeletal injuries during a football career was positively correlated with distress, anxiety and sleeping disturbance, while the number of surgeries was correlated with adverse alcohol behaviour and smoking. Professional footballers who had sustained one or more severe musculoskeletal injuries during their career were two to nearly four times more likely to report symptoms of common mental disorders than professional footballers who had not suffered from severe musculoskeletal injuries.

**Conclusion:**

It can be concluded that the number of severe musculoskeletal injuries and surgeries during a career is positively correlated and associated with symptoms of common mental disorders among male European professional footballers. This study emphasises the importance of applying a multidisciplinary approach to the clinical care and support of professional footballers, especially when a player faces lengthy periods without training and competition as a consequence of recurrent severe joint or muscle injuries.

**Level of evidence:**

III.

## Introduction

During their career, professional footballers (soccer players) are highly at risk of acute , recurrent and severe musculoskeletal injuries [3, 9, 11, 16]; especially, severe musculoskeletal injuries (joint, muscles) can lead to long periods without training or competition, to surgeries and, in the worst case, to early retirement from professional football [6, 9, 16, 17]. In the scientific literature, several risk factors for the occurrence of injuries have been reported, especially physical and environmental factors [9, 11, 12]. In addition, few authors have also suggested that the occurrence of musculoskeletal injuries could be explained by psychological predictors such as somatic and psychic trait anxiety, life event stress, trait irritability and ineffective coping [24, 25, 27].

In contrast to the extensive amount of information available about musculoskeletal injuries, scientific information about the occurrence of symptoms of common mental disorders (CMD) among professional footballers is lacking [17]. Symptoms of CMD can be classified in 20 different domains, which include symptoms related to depression, anxiety, sleep, neurocognition and substance abuse/addiction [2]. The lack of scientific literature about the occurrence of symptoms of CMD in professional football is peculiar because like in elite athletes from other sport disciplines, severe musculoskeletal injuries (joint, muscles) occurring during a sport career can be considered to be major physical and psychosocial stressors that may induce symptoms of CMD [33, 39]. In a previous pilot study, symptoms of distress, anxiety/depression and substance abuse were found to be rather prevalent among current professional players [19]. Consequently, one might assume that the occurrence of these symptoms of CMD among professional footballers might be associated with the occurrence and recurrence of severe joint and muscles injuries sustained during a football career. At the present time, this potential relation of severe musculoskeletal injuries with symptoms of CMD has not been studied among European professional footballers. Such a study is needed in order to explore the need of a multidisciplinary approach to the clinical care and support of professional footballers.

Accordingly, the primary aim of the present study was to explore the associations of severe musculoskeletal injuries (joint, muscle) and surgeries during a football career with symptoms of CMD (distress, anxiety/depression, sleeping disturbance, adverse alcohol behaviour, smoking, adverse nutrition behaviour) among male European professional footballers. The primary hypothesis being tested was that players who had sustained one or more severe musculoskeletal injuries during their career were more likely to report symptoms of CMD than players who had not suffered from severe musculoskeletal injuries during their career.

## Materials and methods

Since April 2014, an international prospective cohort study about CMD in professional football has been conducted in 11 countries across three continents. Being reported in compliance with the STROBE statement, the present study was a cross-sectional analysis of baseline questionnaires from a sample of participants from the international prospective cohort study [36]. Official ethical approval for the study was obtained by the board of St. Marianna University School of Medicine (16 April 2014; Kawasaki, Japan). The present research was conducted in accordance with the Declaration of Helsinki (2013).

**Fig. 1:**
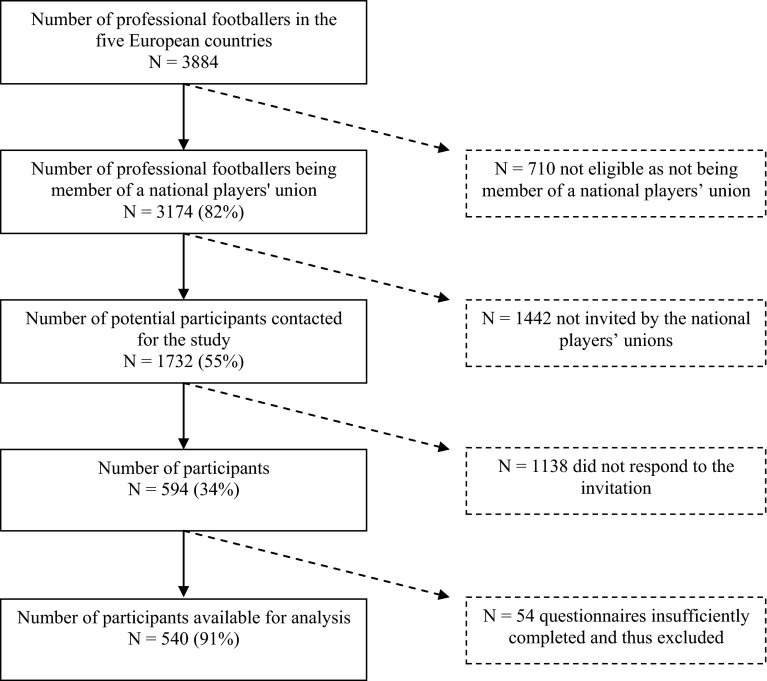
Recruitment of European professional footballers

Participants were professional footballers fulfilling the following inclusion criteria: (1) being a member as an active player of the national players’ union from Finland, France, Norway, Spain or Sweden, which means committing significant time to football training and competing at professional football level; (2) being 18 years old or older; (3) being male; and (4) being able to read and comprehend texts fluently in either English, French or Spanish. In professional football, being a member of a national players’ union is not mandatory but most of the footballers playing in the highest and second highest national leagues are members of their national players’ unions (Fig. 1). Under the assumption based on a previous pilot study that at least one out of ten players might suffer from a health condition under study, sample size calculation indicated that at least 138 participants were needed (confidence interval of 95 %; precision of 5 %) [19, 29].

### Severe musculoskeletal injuries and surgeries (independent variables)

Three questions were used to examine (1) the total number of severe musculoskeletal injuries during a professional football career (e.g. ‘How many severe injuries have you had so far during your professional football career?’), (2) the total number of severe joint injuries during a professional football career and (3) the total number of severe muscle injuries during a professional football career. In our study, severe musculoskeletal injury was defined as an injury that involved the musculoskeletal system (bone, joint, ligament, muscles, tendons…) and occurred during team activities and led to either training or match absence for more than 28 days (definition being clearly stated to the participants) [13]. The total number of surgeries undergone during a professional football career was examined through a single question (e.g. ‘How many surgeries have you had so far during your professional football career?’).

### Symptoms of common mental disorders (dependent variables)

#### Distress

Distress in the previous 4 weeks was measured using the distress screener (three items scored on a 3-point scale) which is based on the Four-Dimensional Symptom Questionnaire (4DSQ) (e.g. ‘Did you recently suffer from worry?’) [4, 34]. The 4DSQ, i.e. distress screener, has been validated in several populations and languages among which English, French and Spanish (internal consistency: 0.6–0.7; test–retest coefficients: ≥0.9; criterion-related validity: area under ROC curve ≥0.8) [4, 34]. A total score ranging from 0 to 6 was obtained by summing up the answers on the three items, a score of 4 or more indicating the presence of distress [4, 34].

#### Anxiety/depression

The 12-item General Health Questionnaire (GHQ-12) was used to assess psychological symptoms related to anxiety/depression in the previous four weeks (e.g. ‘Have you recently felt under strain?’) [14]. The GHQ-12 has been validated in several populations and languages among which English, French and Spanish (internal consistency: 0.7–0.9; criterion-related validity: sensitivity ≥0.7, specificity >0.7, area under ROC curve ≥0.8) [14, 32]. Based on the traditional scoring system, a total score ranging from 0 to 12 was calculated by summing up the answers on the 12 items, with a score of 2 or more indicating the presence of anxiety/depression (area under curve = 0.9) [14].

#### Sleeping disturbance

Derived from the validated PROMIS (short form), sleeping disturbance in the previous 4 weeks was assessed through two single questions (e.g. ‘Did you recently have some problem to sleep?’) scored on a 4-point scale (0 for favourable answers, 1 for unfavourable answers) [40]. The PROMIS has been validated in several populations and languages among which English, French and Spanish (internal consistency: >0.9; construct validity: product-moment correlations ≥0.9) (for detailed information, see www.nihpromis.org) [5, 40]. A total score ranging from 0 to 2 was obtained by summing up the answers to the two questions, a score of 1 or more indicating the presence of sleep disturbance [40].

#### Adverse alcohol behaviour

Current level of alcohol consumption was detected using the validated 3-item AUDIT-C (e.g. ‘How many standard rinks containing alcohol do you have on a typical day?’) [7]. The AUDIT-C has been validated in several populations and languages among which English, French and Spanish (test–retest coefficients: 0.6–0.9; criterion-related validity: area under ROC curve 0.70–< 1.0) [7, 8]. A total score ranging from 0 to 12 was obtained by summing up the answers on the three items, a score of 5 or more indicating the presence of adverse alcohol behaviour [7].

#### Smoking

Current smoking behaviour (tobacco) was assessed through a single question (e.g. ‘Do you smoke cigarettes’) (yes or no).

#### Adverse nutrition behaviour

Current eating habits were examined using four simple statements (validated in English and translated for the study in French and Spanish), each to be answered by how many days per week (from 0 to 7) this is the case (e.g. ‘I eat regularly throughout the day’) [35]. Consuming healthy meals less than 5 days per week and eating regularly throughout the day less than 3 days per week and having breakfast before 10:30 less than 3 days per week and having a final meal before 20:30 less than 3 days per week was reported as adverse nutrition behaviour [35].

### Procedures

Based on the dependent (symptoms of CMD) and independent (injuries and surgeries) variables included in the study, an electronic anonymous questionnaire available in English, French and Spanish was set up (FluidSurveys™). In addition, the following descriptive variables were retrieved: age, length, body mass, duration of professional football career, level of play and squad position. The World Players’ Union (FIFPro)—representing over 65,00 professional footballers worldwide across all continents—asked the national players’ unions in Finland, France, Norway, Spain and Sweden to identify potential participants from their members according to the inclusion criteria mentioned previously. With regard to a low response rate observed in a previous pilot study, FIFPro asked the five national players’ unions to invite as many potential participants as possible [19]. Information about the study was sent per email to potential participants by the national players’ unions, recruitment of participants lasting from April to September 2014 and invitation procedures being blinded to the responsible researchers for reasons of privacy and confidentiality of the players. If they were interested in participating in the study, participants gave their informed consent and were asked to complete their questionnaires anonymously online within 2 weeks (two reminders sent after 2 and 4 weeks). Once completed (around 15 min was required), the electronic anonymous questionnaires were saved automatically on a secured electronic server that was accessible only by the principal researcher. Players participated voluntarily in the study and did not receive any reward for their participation.

### Statistical analyses

All data analyses were performed using the statistical software IBM SPSS Statistics 22.0 for Windows. Only questionnaires sufficiently completed were eligible for analyses: at least 50 % of the descriptive variables and at least 50 % of the outcome measures needed to be fully completed without missing data (no imputation). Descriptive data analyses (mean, standard deviation, frequency, range) were performed for all variables included in our study, while prevalences of symptoms of distress, anxiety/depression, sleep disturbance, adverse alcohol behaviour, smoking and adverse nutrition behaviour were calculated using the Wald method for 95 % confidence interval (95 % CI) [31]. Pearson correlation coefficients were used to explore the direction and relative strength of a potential relationship of severe musculoskeletal injuries and surgeries (continuous variable) with symptoms of CMD (continuous variable) [31]. Univariate logistic regression analyses (expressed as odds ratio OR and related 95 % CI) were used to explore whether professional footballers who had sustained one, two and three or more severe musculoskeletal injuries or surgeries (independent variable in categories: 0, 1, 2 and ≥3) during their career were more likely to report symptoms of CMD (dichotomous dependent variable: presence or absence) than professional footballers who had not [31].

## Results

### Participants and prevalences

The flow diagram of the respondents’ recruitment is presented in Fig. 1. The national players’ unions of Finland, France, Norway, Spain and Sweden contacted a sample of 1732 professional footballers, 594 of whom gave their written informed consent (overall response rate of 34 %). As 54 questionnaires were insufficiently completed, 540 professional footballers were included in the analyses (mean age of 27 years; 54 % playing in the highest leagues). During their football career (mean duration of 8 years), the players had already sustained on average 2.3 severe musculoskeletal injuries (SD = 2.4), 1.4 severe joint injuries (SD = 1.6) and 1.4 severe muscle injuries (SD = 2.0), and they had undergone on average 1.1 surgeries (SD = 1.4). Of the respondents, 79 % had incurred one or more severe musculoskeletal injuries during their career, 68 % one or more severe joint injuries and 60 % one or more severe muscle injuries, while 54 % had undergone one or more surgeries. Prevalence of symptoms of CMD ranged from 3 % for smoking and 10 % for adverse alcohol behaviour to 37 % for anxiety/depression and 58 % for adverse nutrition behaviour. Descriptives of the respondents and prevalence rates are presented in Table 1.

**Table 1:** Descriptives (reported as mean ± SD, or percentage) and prevalence of symptoms of CMDs among European professional footballers (*N* = 540)

Variables		
Age (in years)	26.7	±4.4
Height (in cm)	181.4	±7.4
Weight (in kg)	76.8	±8.3
Duration of the professional football career (in years)	7.8	±4.4
Level of play (top league)	53.5	
*Field position*		
Goalkeeper	13.5	
Defender	38.0	
Midfielder	31.9	
Forward	16.7	
*Average number of severe injuries and surgeries*		
All severe injuries	2.3	±2.4
Severe joint injuries	1.4	±1.6
Severe muscle injuries	1.4	±2.0
Surgeries	1.1	±1.4
*Prevalence of symptoms of CMD*		
Distress^a^	15.1	(95 % CI 11.9–18.3)
Anxiety/depression^a^	37.3	(95 % CI 32.8–41.9)
Sleeping disturbance^a^	24.0	(95 % CI 20.2–27.8)
Adverse alcohol behaviour^b^	10.3	(95 % CI 7.5–13.0)
Smoking^b^	3.2	(95 % CI 1.6–4.8)
Adverse nutritional behaviour^b^	58.2	(95 % CI 53.8–62.7)

*N* number of participants, *SD* standard deviation, *cm* centimetres, *kg* kilograms, *CI* confidence interval

^a^One-month prevalence

^b^Point prevalence

### Correlations and associations

Professional footballers who had sustained three or more severe musculoskeletal injuries during their career were more than two times more likely to report distress (OR 2.7 and 95 % CI 1.2–5.9) and sleeping disturbance (OR 2.3 and 95 % CI 1.2–4.4) than professional footballers who had not suffered from severe musculoskeletal injuries during their career. Professional footballers who had sustained two severe musculoskeletal injuries during their career were nearly three and a half times more likely to report adverse alcohol behaviours (OR 3.4 and 95 % CI 1.3–8.9) than professional footballers who had not suffered from severe musculoskeletal injuries during their career. Professional footballers who had sustained one or more severe joint injuries during their career were three to nearly four times more likely to report distress (OR 3.8 and 95 % CI 1.7–8.4), sleeping disturbance (OR 3.4 and 95 % CI 1.9–6.2) and adverse alcohol behaviour (OR 3.4 and 95 % CI 1.4–8.2) than professional footballers who had not suffered from severe joint injuries during their career. Professional footballers who had sustained three or more severe muscle injuries during their career were two and a half times more likely to report distress (OR 2.6 and 95 % CI 1.37–4.9) than professional footballers who had not suffered from severe muscle injuries during their career. There was also a significant association of one or more severe joint injuries or surgeries during professional football career with adverse nutritional behaviour. All correlations found in our study were weak. All associations (OR and 95 % CI) are presented in Table 2.

**Table 2:** Associations (odds ratio and 95 % confidence interval) of severe injuries and surgeries with symptoms of CMDs among European professional footballers (*N* = 540)

		Distress	Anxiety/depression	Sleeping disturbance	Adverse alcohol behaviour	Smoking	Adverse nutrition behaviour
Severe injuries	0	1.00	1.00	1.00	1.00	1.00	1.00
	1	1.36 (0.56–3.29)	0.73 (0.40–1.32)	1.68 (0.84–3.38)	1.28 (0.44–3.75)	1.05 (0.28–4.04)	1.09 (0.63–1.90)
	2	1.84 (0.77–4.38)	1.29 (0.72–2.29)	1.99 (0.98–4.01)	**3.40 (1.29**–**8.92)**	0.69 (0.15–3.16)	1.13 (0.64–1.99)
	≥3	**2.69 (1.23**–**5.87)**	1.12 (0.65–1.91)	**2.32 (1.22**–**4.42)**	1.44 (0.54–3.89)	0.44 (0.10–2.01)	1.03 (0.62–1.72)
Severe joint injuries	0	1.00	1.00	1.00	1.00	1.00	1.00
	1	**2.95 (1.37**–**6.37)**	1.15 (0.70–1.87)	1.73 (0.98–3.07)	**3.37 (1.39**–**8.15)**	0.96 (0.27–3.38)	**0.49 (0.31**–**0.79)**
	2	**3.78 (1.69**–**8.42)**	1.30 (0.75–2.26)	**3.41 (1.89**–**6.17)**	**2.97 (1.14**–**7.75)**	0.87 (0.20–3.73)	0.70 (0.41–1.19)
	≥3	**3.50 (1.51**–**8.12)**	1.18 (0.65–2.14)	1.31 (0.65–2.64)	1.62 (0.52–5.00)	0.73 (0.14–3.83)	0.77 (0.43–1.36)
Severe muscle injuries	0	1.00	1.00	1.00	1.00	1.00	1.00
	1	1.34 (0.73–2.46)	0.82 (0.52–1.29)	1.29 (0.79–2.11)	1.82 (0.93–3.58)	2.20 (0.72–6.70)	0.68 (0.44–1.04)
	2	0.70 (0.23–2.15)	1.36 (0.69–2.66)	0.90 (0.40–2.02)	0.78 (0.22–2.81)	0.85 (0.10–7.43)	1.44 (0.71–2.89)
	≥3	**2.55 (1.32**–**4.92)**	0.56 (0.30–1.04)	1.53 (0.85–2.76)	0.88 (0.33–2.34)	–	1.22 (0.71–2.11)
Surgeries	0	1.00	1.00	1.00	1.00	1.00	1.00
	1	1.20 (0.66–2.19)	1.05 (0.65–1.70)	1.22 (0.73–2.04)	1.82 (0.88–3.75)	2.19 (0.72–6.67)	0.85 (0.54–1.35)
	2	0.83 (0.36–1.89)	0.85 (0.46–1.59)	1.51 (0.81–2.81)	1.46 (0.58–3.70)	0.57 (0.07–4.78)	0.62 (0.35–1.10)
	≥3	1.01 (0.48–2.12)	0.99 (0.56–1.75)	1.07 (0.57–2.00)	1.51 (0.62–3.67)	0.51 (0.06–4.29)	**0.42 (0.25**–**0.73)**

*N* number of participants

Bold values indicate significant *P* values (*P* ≤ 0.01)

## Discussion

The principal findings of the present study were that: (1) prevalences of symptoms of CMD among male European professional footballers ranged from 3 % for smoking to 37 % for anxiety/depression and 58 % for adverse nutrition behaviour; (2) the number of severe musculoskeletal injuries during football career was positively correlated with symptoms of distress, anxiety/depression and sleeping disturbance; and (3) male professional footballers who had sustained one or more severe musculoskeletal injuries (joint or muscle) during their career were two to nearly four times more likely to report symptoms of distress, sleeping disturbance or adverse alcohol behaviour than male professional footballers who had not suffered from severe musculoskeletal injuries during their career.

### Perspective of the findings

In 2013, FIFPro conducted a pilot study on the psychosocial health problems among professional footballers from Australia, Ireland, the Netherlands, New Zealand, Scotland and USA [19]. In this study, the assumption was made that the odds of suffering from psychosocial health problems may be greater for players more exposed to severe musculoskeletal injuries (time loss of more than 28 days) and surgeries compared to players less unexposed to these stressors [19]. Despite the small sample of participants (*N* = 149), results showed that a higher number of severe musculoskeletal injuries and surgeries among professional footballers were significantly correlated with symptoms of distress, anxiety/depression and adverse health behaviour, which is in line with the results found in our study [19]. The present study found that male professional footballers who had sustained one or more severe musculoskeletal injuries (joint or muscle) during their career were two to nearly four times more likely to report symptoms of CMD than male professional footballers who had not suffered from severe musculoskeletal injuries during their career. Furthermore, post hoc analyses (univariate logistic binary regression with number of injuries as continuous independent variable) showed that European professional footballers were 10–25 % more likely to report symptoms of distress or sleeping disturbance by every additional severe (joint or muscle) injury (*p* ≤ 0.01). As far as the authors know, there is no other scientific information in international professional football about the influence of physical stressors such as injuries or surgeries on symptoms of CMD, while the influence of symptoms of CMD on the occurrence of injuries has been barely studied. Recently, Gulliver et al. [23] found that injured Australian athletes had significantly higher symptoms of depression and general anxiety than non-injured athletes, which is partly in line with our findings.

In the UEFA Champions League, it was shown that the rate of overall severe injury from 2001 to 2012 did not change over 11 seasons, while knee joint injuries accounted for 6 % of all time-loss injuries (14 % of which were anterior cruciate ligament injuries) [10, 37, 38]. Between 2008 and 2013 in the Australia A-League, knee and ankle severe injuries were responsible for 180–500 official matches missed per season [16]. Consequently, with regard to the recurrence of severe musculoskeletal injuries during a football career, it does not seem strange that some high prevalences of symptoms of CMD were found in our study sample. In a latest study performed on the UEFA Champions League data from 2001 to 2012, Nordström et al. [30] showed that professional footballers that suffered from concussions had more injuries before their (first) concussion than players that did not suffer from concussions. In addition, the authors showed that these concussed players had a higher risk of injuries up to a year after the concussion compare to the non-concussed players [30]. Analogously, and in the light of our findings, we could hypothesize that severely injured professional footballers that are reporting symptoms of CMD might be subsequently even more at risk of injuries than players without symptoms of CMD. However, such an assumption has not been studied yet.

### Methodological considerations

Several strengths and limitations of our study should be pointed out. The major strengths of our study rely in the topic being covered in a large number of male professional footballers. Despite the initiatives of several national players’ unions such as in England and France, CMD remains taboo in professional football, where attention is principally given to physical health aspects [1, 17]. Despite the fact that the 540 participants (54 % of whom were playing in the highest division in their country) involved in the present study represent around 15 % of the total number of professional footballers in Finland, France, Norway, Spain and Sweden, their involvement should significantly contribute to raising the self-awareness within professional football about the occurrence of symptoms of CMD, which seems to be a prerequisite before the development and implementation of supportive and preventive measures.

The principal limitation of the present study is the cross-sectional analyses conducted on our data. Such a cross-sectional design does not allow the establishment of a causal relationship between independent (severe musculoskeletal injuries and surgeries) and dependent (symptoms of CMD) variables but only enable us to explore their potential associations [31]. Another potential limitation might be the response rate achieved in our study. Despite the fact that professional footballers were informed about the study and invited to participate by their national players’ unions, only one out of three players was willing to be involved in the study, which was lower than we expected. In addition, as the recruitment procedures were blind to the researchers for privacy and confidentially reasons, non-response analysis could not be performed. With regard to the validity of the data collected, a potential limitation worth mentioning is directed towards the screening instrument used to explore adverse nutrition behaviour. By contrast to the scales used for the other outcome measures (distress screener, GHQ-12, PROMIS, AUDIT-C), the four statements related to nutrition have not been validated in French and Spanish (simply translated). Also, one might argue that information about severe musculoskeletal injuries was self-reported and not gathered according to the scientifically accepted consensus statement on data collection procedures in football [13]. In addition, the retrospective recall of unpleasant experiences in the past such as severe musculoskeletal injury might be questionable. Two defence mechanisms, denial and repression, are well known for influencing retrospective recall: denial refers to the deny of admitting that something unpleasant has occurred, while repression acts to keep information out of conscious awareness [26]. From our study population (professional footballers), we know that players can generally remember quite precisely the number of severe musculoskeletal injuries they suffered and that led to at least 4 weeks without training or competition. However, as severe musculoskeletal injuries can be considered as major life events during a professional football career, we cannot categorically exclude that participants were unable to recall all the injuries during their entire career. In our study, involving clubs by using their medical records for injury during an entire football career was not feasible, especially because players might have not want to report any CMD when clubs would have been involved in the study. Also, we believe that the use of electronic questionnaires enabled us to guarantee the consistency of data collection among so many participants from five different European countries.

### Implications

The findings of our study might contribute largely to raising the self-awareness of the different stakeholders in professional football (such as clubs, players’ unions or Union of European Football Associations) about the occurrence of symptoms of CMD among players and its relation with severe musculoskeletal injuries. Also, our study emphasises the need to develop and implement supportive and preventive measures. Recently, both current and retired professional footballers as well as their club physicians acknowledged that the medical care and support as well as the medical examinations during a football career were exclusively directed towards physical health (mostly injuries), while adequate support related to psychosocial well-being was reported to be lacking and necessary [1, 18]. In terms of support related to psychosocial well-being, Gulliver et al. [21] showed that raising self-awareness about the occurrence of symptoms of CMD among athletes, access to internet-based interventions, and the positive attitudes of all stakeholders (especially coaches) were reported as facilitators to help-seeking.

The clinical relevance of this study is that it emphasise the importance of applying a multidisciplinary approach to the clinical care and support of professional footballers, especially when a player faces lengthy periods without training and competition as a consequence of recurrent severe joint or muscle injuries; especially, severe joint injuries and related surgeries should be monitored across clubs in the long term, not only because these have been recognized as risk factors for the development of osteoarthritis in the post-sport life, but also because of their potential consequences in terms of symptoms of CMD as shown in the present study [15, 20, 28]. In order to protect and promote the sustainable health of professional footballers, supportive and preventive evidence-based measures directed towards symptoms of CMD should be developed and implemented in professional football, especially online interventions based on a self-management approach [17, 22]. Also, further research is needed, especially research based on longitudinal design, that allows gaining insight into the causal relationship between risk factors and symptoms of CMD (as included in the present study or other symptoms). Such a longitudinal design might also enable an exploration of the sequence of being severely injured, reporting symptoms of CMD, and being even more at risk for the occurrence of injuries in the subsequent period.

## Conclusion

It can be concluded that the number of severe musculoskeletal injuries (joint and muscle) and surgeries during a career is positively correlated and associated with symptoms of CMD among male European professional footballers.

## References

[1] Akturk A, Inklaar H, Gouttebarge V, Frings-Dresen MHW (2014). Medical examinations in the Dutch professional football (soccer): a qualitative study. Int SportMed J.

[2] American Psychiatric Association (2000). Diagnostic and statistical manual of mental disorders.

[3] Aoki H, O’Hata N, Kohno T, Morikawa T, Seki J (2012). A 15-year prospective epidemiological account of acute traumatic injuries during official professional soccer league matches in Japan. Am J Sports Med.

[4] Braam C, van Oostrom SH, Terluin B, Vasse R, de Vet HC, Anema JR (2009). Validation of a distress screener. J Occup Rehabil.

[5] Buysse DJ, Yu L, Moul DE, Germain A, Stover A, Dodds NE, Johnston KL, Shablesky-Cade MA, Pilkonis PA (2010). Development and validation of patient-reported outcome measures for sleep disturbance and sleep-related impairments. Sleep.

[6] Chen SK, Cheng YM, Lin YC, Hong YJ, Huang PJ, Chou PH (2005). Investigation of management models in elite athlete injuries. Kaohsiung J Med Sci.

[7] Dawson DA, Grant BF, Stinson FS, Zhou Y (2005). Effectiveness of the derived alcohol use disorders identification test (AUDIT-C) in screening for alcohol use disorders and risk drinking in the general population. Alcohol Clin Exp Res.

[8] De Meneses-Gaya C, Waldo Zuardi A, Loureiro SR, Crippa JAS (2009). Alcohol use disorders identification test (AUDIT): an updated systematic review of psychometric properties. Psychol Neurosci.

[9] Ekstrand J, Waldén M, Hägglund M (2004). A congested football calendar and the wellbeing of players: correlation between match exposure of European footballers before the World Cup 2002 and their injuries and performances during that World Cup. Br J Sports Med.

[10] Ekstrand J, Hägglund M, Kristenson K, Magnusson H, Waldén M (2013). Fewer ligament injuries but no preventive effect on muscle injuries and severe injuries: an 11-year follow-up of the UEFA champions league injury study. Br J Sports Med.

[11] Ekstrand J, Hägglund M, Waldén M (2011). Injury incidence and injury patterns in professional football: the UEFA injury study. Br J Sports Med.

[12] Fuller CW, Junge A, Dvorak J (2012). Risk management: FIFA’s approach for protecting the health of football players. Br J Sports Med.

[13] Fuller CW, Ekstrand J, Junge A, Andersen TE, Bahr R, Dvorak J, Hägglund M, McCrory P, Meeuwisse WH (2006). Consensus statement on injury definitions and data collection procedures in studies of football (soccer) injuries. Br J Sports Med.

[14] Goldberg DP, Gater R, Sartorius N, Ustun TB, Piccinelli M, Gureje O (1997). The validity of two versions of the GHQ in the WHO study of mental illness in general health care. Psychol Med.

[15] Gouttebarge V, Frings-Dresen MHW (2014) Ankle osteoarthritis in former professional football players: what do we know? In: d’Hooghe P, Kerkhoffs G (ed) The ankle in football. Springer, Heidelberg, pp 311–322

[16] Gouttebarge V, Hughes Schwab BA, Vivian A, Kerkhoffs G (2015) Injuries, match missed and effect of minimum medical standards in the A-LEAGUE professional football: a 5-year prospective study. Asian J Sports Med Accepted for publication 10.5812/asjsm.31385 PMC4870823 27217935

[17] Gouttebarge V, Aoki H (2014). Life span perspective of professional footballers’ health. Asian J Sports Med.

[18] Gouttebarge V, Sluiter JK (2013). Medical examinations in the Dutch professional football. Occup Med.

[19] Gouttebarge V, Frings-Dresen MHW, Sluiter JK (2015). Mental and psychosocial health among current and former professional football players. Occup Med.

[20] Gouttebarge V, Inklaar H, Frings-Dresen MHW (2014). Risk and consequences of osteoarthritis after a professional football career: a systematic review of the recent literature. J Sports Med Phys Fitness.

[21] Gulliver A, Griffiths KM, Christensen H (2012). Barriers and facilitators to mental health help-seeking for young elite athletes: a qualitative study. BMC Psychiatry.

[22] Gulliver A, Griffiths KM, Christensen H, Mackinnon A, Calear AL, Parsons A, Bennett K, Batterham PJ, Stanimirovic R (2012). Internet-based interventions to promote mental health help-seeking in elite athletes: an exploratory randomized controlled trial. J Med Internet Res.

[23] Gulliver A, Griffiths KM, Mackinnon A, Batterham PJ, Stanimirovic R (2015). The mental health of Australian elite athletes. J Sci Med Sport.

[24] Ivarsson A, Johnson U (2010). Psychological factors as predictors of injuries among senior soccer players. A prospective study. J Sports Sci Med.

[25] Ivarsson A, Johnson U, Podlog L (2013). Psychological predictors of injury occurrence: a prospective investigation of professional Swedish soccer players. J Sport Rehabil.

[26] Jacobson E (1957). Denial and repression. J Am Psychoanal Assoc.

[27] Johnson U, Ivarsson A (2011). Psychological predictors of sport injuries among junior soccer players. Scand J Med Sci Sports.

[28] Kuijt M-TK, Inklaar H, Gouttebarge V, Frings-Dresen MHW (2012). Knee and ankle osteoarthritis in former elite soccer players: a systematic review of the recent literature. J Sci Med Sport.

[29] Lwanga SK, Lemeshow S (1991). Sample size determination in health studies. A practical manual.

[30] Nordström A, Nordström P, Ekstrand J (2014). Sport-related concussion increases the risk of subsequent injury by about 50% in elite male football players. Br J Sports Med.

[31] Portney LG, Watkins MP (2008). Foundations of clinical research. Applications to practice.

[32] Salama-Younes M, Montazeri A, Ismaïl A, Roncin C (2009). Factor structure and internal consistency of the 12-item General Health Questionnaire (GHQ-12) and the Subjective Vitality Scale (VS), and the relationship between them: a study from France. Health Qual Life Outcomes.

[33] Shuer ML, Dietrich MS (1997). Psychological effects of chronic injury in elite athletes. West J Med.

[34] Terluin B, Van Marwijk HWJ, Adèr HJ, De Vet HCW, Penninx BWJH, Hermens MLM, Van Boeijen CA, Van Balkom AJLM, Van der Klink JJL, Stalman WAB (2006). The Four-Dimensional Symptom Questionnaire (4DSQ): a validation study of a multidimensional self-report questionnaire to assess distress, depression, anxiety and somatization. BMC Psychiatry.

[35] Van der Veer T, Frings-Dresen MHW, Sluiter JK (2011). Health behaviors, care needs and attitudes towards self-prescription: a cross-sectional survey among Dutch medical students. PLoS One.

[36] Vandenbroucke JP, von Elm E, Altman DG, Pocock SJ, Gøtzsche PC, Vandenbroucke JP, Initiative STROBE (2007). Strengthening the reporting of observational studies in epidemiology (STROBE). Epidemiology.

[37] Waldén M, Hägglund M, Magnusson H, Ekstrand J (2011). Anterior cruciate ligament injury in elite football: a prospective three-cohort study. Knee Surg Sports Traumatol Arthrosc.

[38] Waldén M, Hägglund M, Ekstrand J (2013). Time-trends and circumstances surrounding ankle injuries in men’s professional football: an 11-year follow-up of the UEFA Champions League injury study. Br J Sports Med.

[39] Walker N, Thatcher J, Lavallee D (2007). Psychological responses to injury in competitive sport: a critical review. J R Soc Promot Health.

[40] Yu L, Buysse DJ, Germain A, Moul DE, Stover A, Dodds NE, Johnston KL, Pilkonis PA (2011). Development of short forms from the PROMIS sleep disturbance and sleep-related impairment item banks. Behav Sleep Med.

